# Supplementary material for: A larval zebrafish model of traumatic brain injury: optimizing the dose of neurotrauma for discovery of treatments and aetiology
Source: Biol Open. 2025 Feb 12;14(2):bio060601. doi: 10.1242/bio.060601 (PMC11849975; doi:10.1242/bio.060601)
Supplement: Supplementary information [file biolopen-14-060601-s1.pdf]

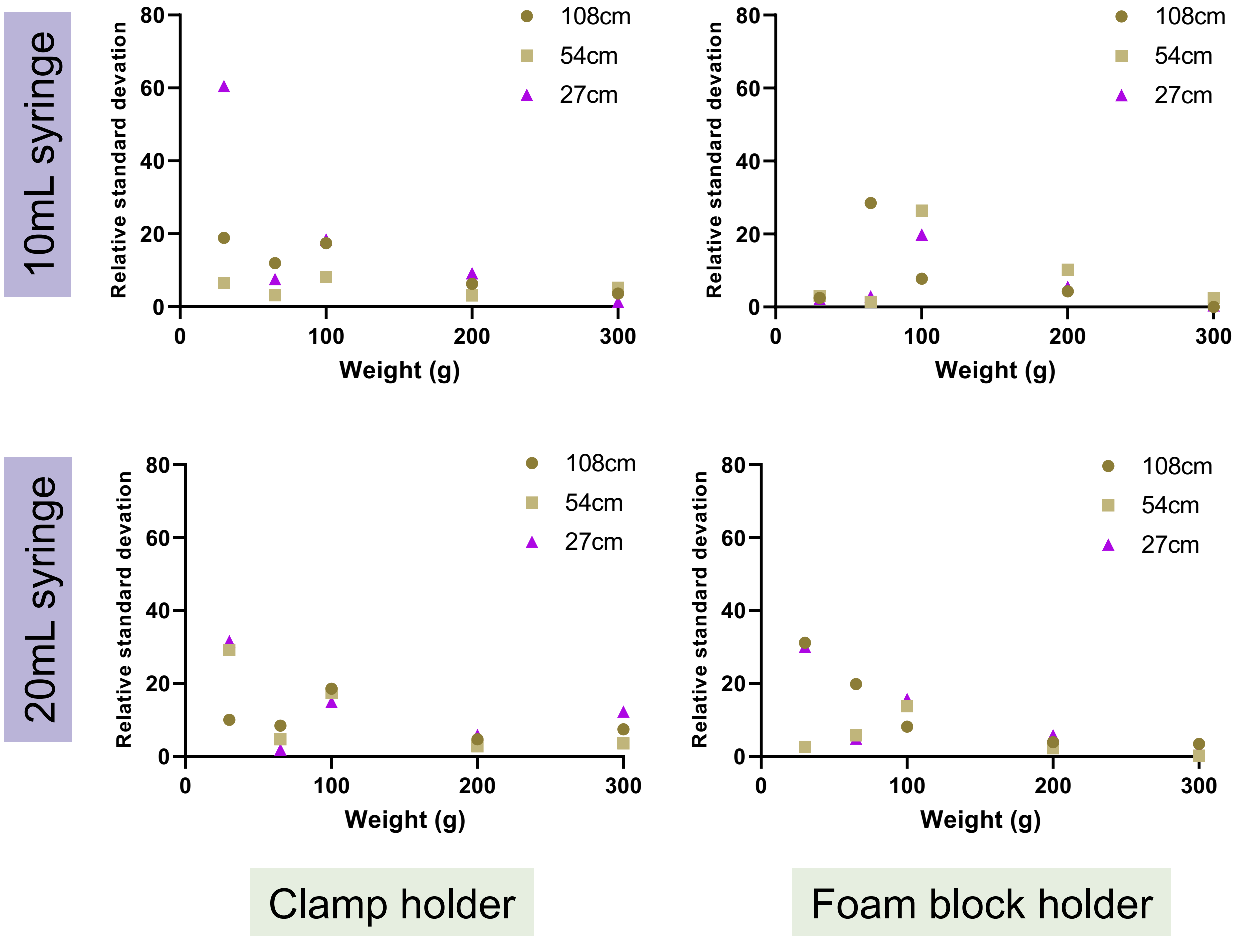

Fig. S1. Relative standard deviation of different TBI assay setups. Relative standard deviation (RSD) was calculated by calculating the percent of the standard deviation value to the mean ([SD/Mean]*100). Weights of higher mass had a lower RSD relative to weights of lower mass. The 65g weight which had a diameter similar to the guide tube diameter had a lower RSD relative to the 35g and 100g weight, which were much smaller than the guide tube diameter in comparison.

Biology Open • Supplementary information

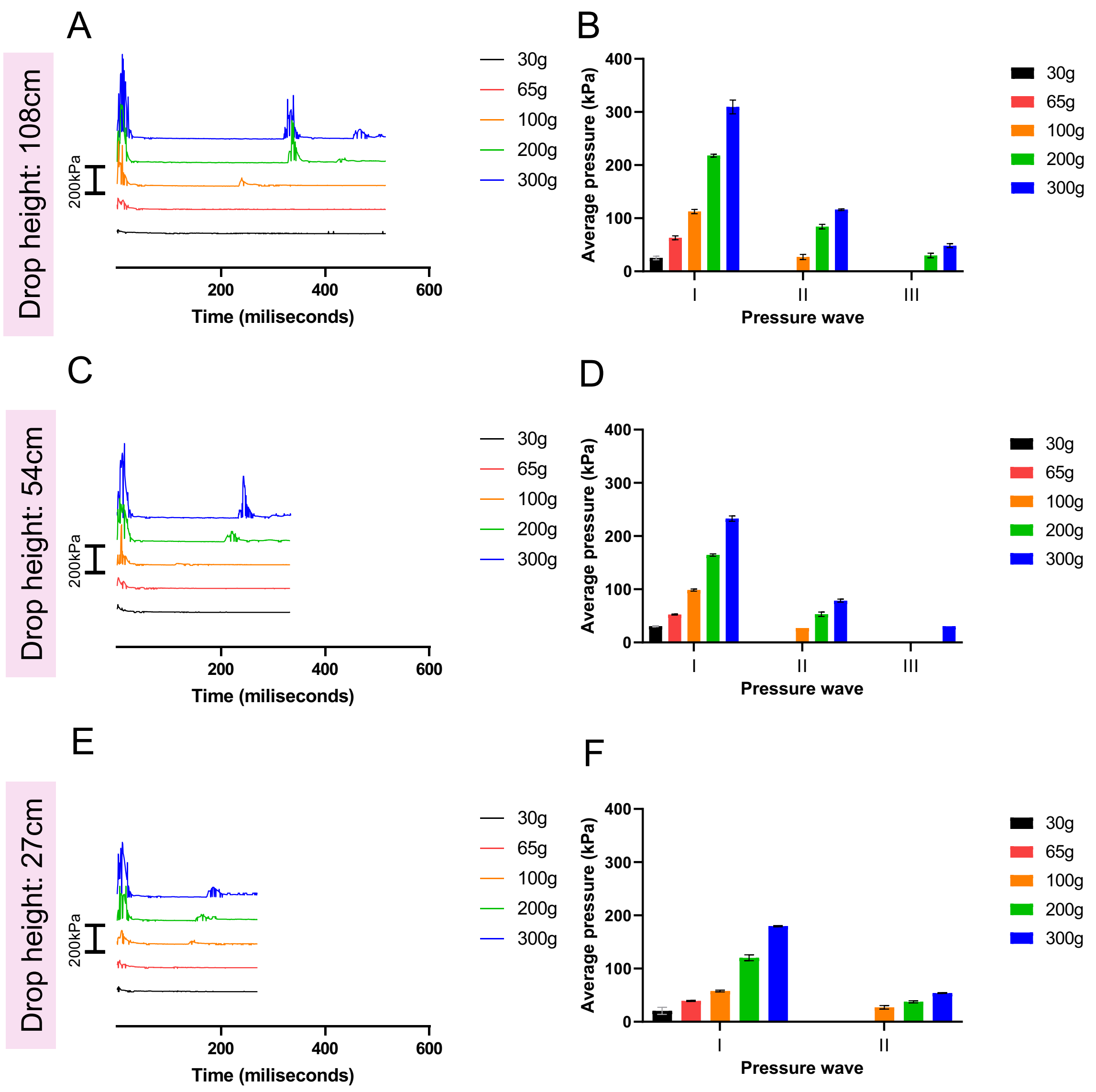

Fig. S2. Characteristics of the pressure waves created in the 10mL syringe held by a clamp TBI model setup. A 10mL syringe was held by a clamp underneath tubes of various heights (108cm, 54cm, 27cm). The syringe tip was attached to a fuel pressure transducer via a 3D-printed Leur-Lok adaptor. Weights ranging from 65-300g were dropped onto the syringe and pressure levels were measured using Arduino IDE software. The data in B, D, F presented represents the average pressure per pressure wave, which was the average of the first time point in milliseconds where pressure rose above zero to the last time point where pressure remained above zero, for each pressure wave. For the time course graphs of each weight, the data is staggered so a reference bar on the x-axis which denotes 200kPa is available. Representative time course of pressure wave dynamics over 600 milliseconds for weights dropped from A) 108cm, C) 54cm, and E) 27cm. Average pressure of each pressure wave (N = 3 weight drops for each weight) for drop heights of B) 108cm, D) 54cm, and F) 27cm.

Biology Open • Supplementary information

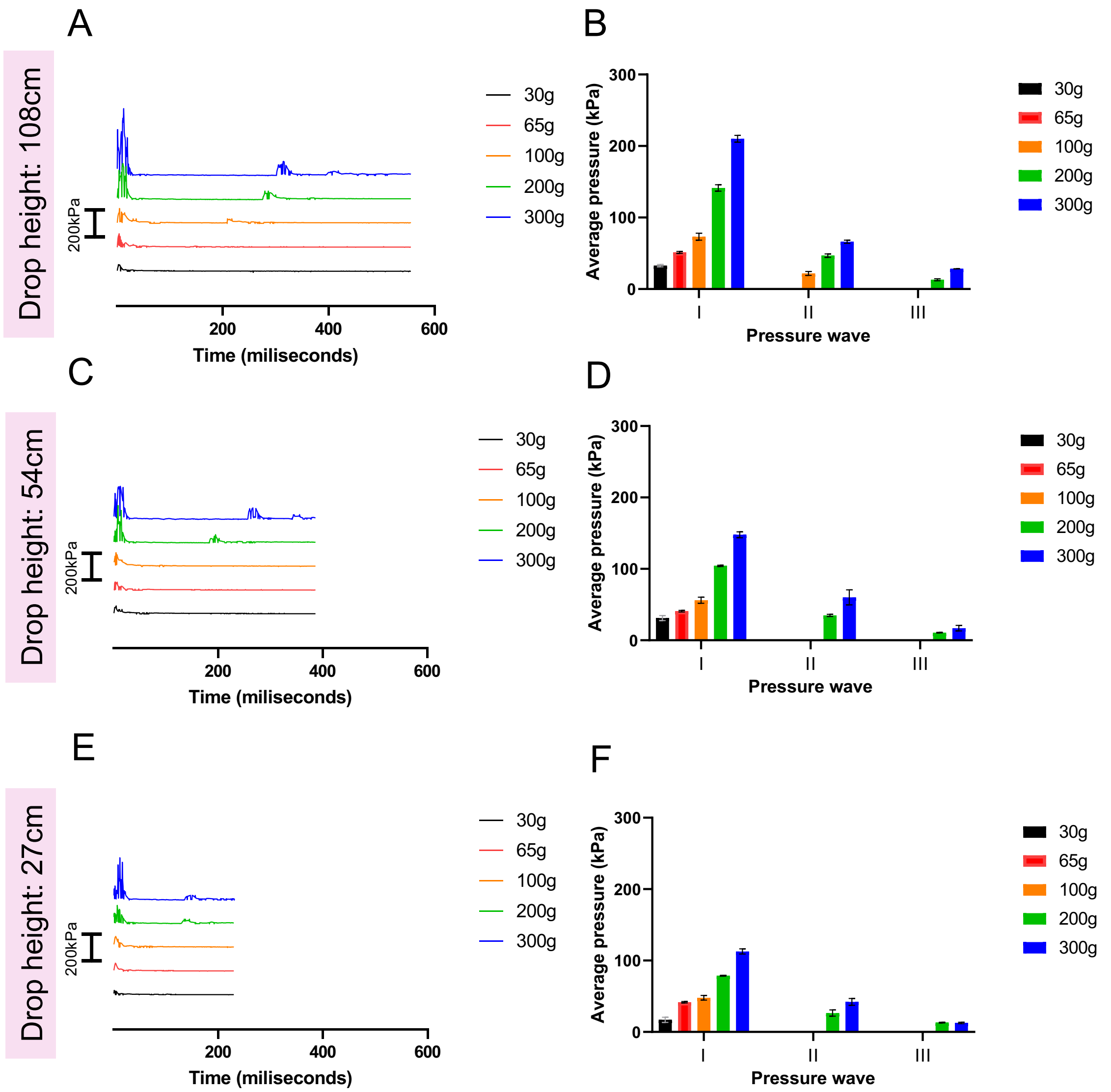

Fig. S3. Characteristics of the pressure waves created in the 20mL syringe held by a clamp TBI model setup.

A 20mL syringe was held by a clamp underneath tubes of various heights (108cm, 54cm, 27cm). The syringe tip was attached to a fuel pressure transducer via a 3D-printed Leur-Lok adaptor. Weights ranging from 65-300g were dropped onto the syringe and pressure levels were measured using Arduino IDE software. The data presented represents the average pressure per pressure wave, which was the average of the first time point in milliseconds where pressure rose above zero to the last time point where pressure remained above zero, for each pressure wave. For the time course graphs of each weight, the data is staggered so a reference bar on the x-axis which denotes 200kPa is available. Representative time course of pressure wave dynamics over 600 milliseconds for weights dropped from A) 108cm, C) 54cm, and E) 27cm. Average pressure of each pressure wave (N = 3 weight drops for each weight) for drop heights of B) 108cm, D) 54cm, and F) 27cm.

Biology Open • Supplementary information

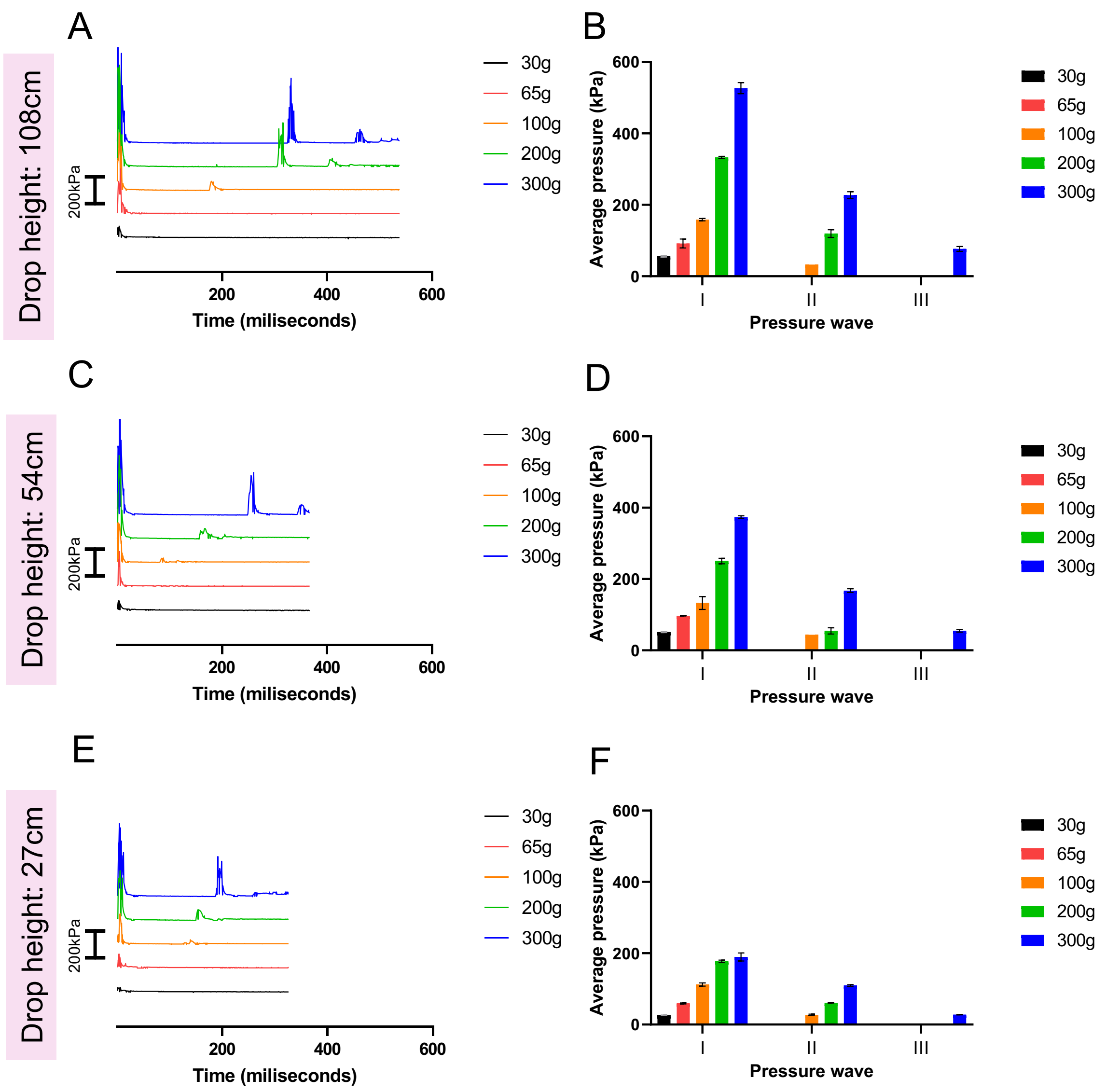

Fig. S4. Characteristics of the pressure waves created in the 10mL syringe held by a foam block TBI model setup. A 10mL syringe was held by a foam block underneath tubes of various heights (108cm, 54cm, 27cm). The syringe tip was attached to a fuel pressure transducer via a 3D-printed Leur-Lok adaptor. Weights ranging from 65-300g were dropped 6onto the syringe and pressure levels were measured using Arduino IDE software. The data presented represents the average pressure per pressure wave, which was the average of the first time point in milliseconds where pressure rose above zero to the last time point where pressure remained above zero, for each pressure wave. For the time course graphs of each weight, the data is staggered so a reference bar on the x-axis which denotes 200kPa is available. Representative time course of pressure wave dynamics over 600 milliseconds for weights dropped from A) 108cm, C) 54cm, and E) 27cm. Average pressure of each pressure wave (N = 3 weight drops for each weight) for drop heights of B) 108cm, D) 54cm, and F) 27cm.

Biology Open • Supplementary information

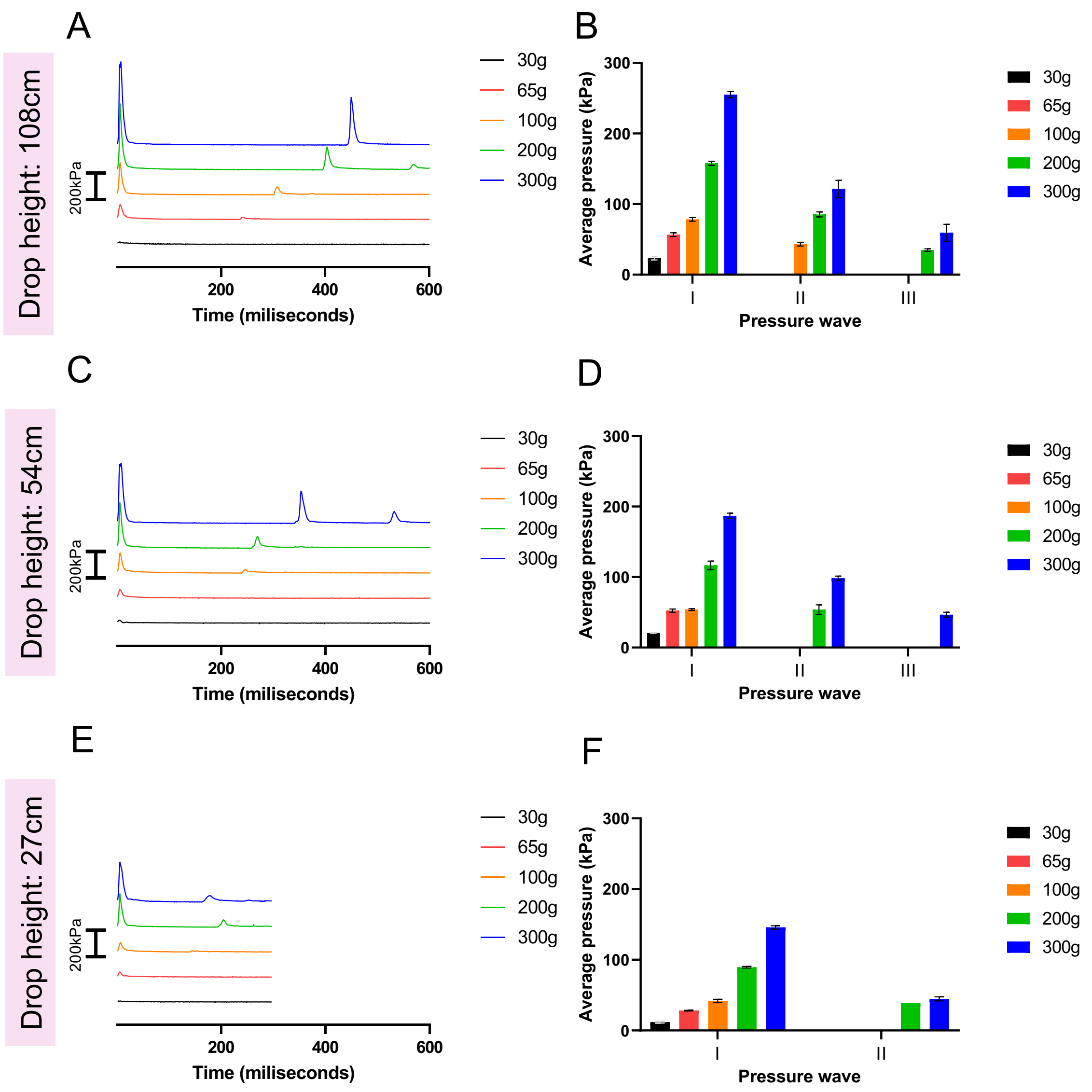

Fig. S5. Characteristics of the pressure waves created in the 20mL syringe held by a foam block TBI model setup. A 20mL syringe was held by a foam block underneath tubes of various heights (108cm, 54cm, 27cm). The syringe tip was attached to a fuel pressure transducer via a 3D-printed Leur-Lok adaptor. Weights ranging from 65-300g were dropped onto the syringe and pressure levels were measured using Arduino IDE software. The data presented represents the average pressure per pressure wave, which was the average of the first time point in milliseconds where pressure rose above zero to the last time point where pressure remained above zero, for each pressure wave. For the time course graphs of each weight, the data is staggered so a reference bar on the x-axis which denotes 200kPa is available. Representative time course of pressure wave dynamics over 600 milliseconds for weights dropped from A) 108cm, C) 54cm, and E) 27cm. Average pressure of each pressure wave (N = 3 weight drops for each weight) for drop heights of B) 108cm, D) 54cm, and F) 27cm.

Table S1. Average pressure values measured in the 10mL syringe held by the clamp mount

Drop height (cm)	Mass of weight (g)	Average maximal pressure (kPa)	Average pressure 1st wave (kPa)	Average pressure 2nd wave (kPa)	Average pressure 3rd wave (kPa)
108	30	35.38	27.38	N/A	N/A
	65	108.3	63.06	N/A	N/A
	100	203.9	112.7	26.98	N/A
	200	502.6	217.8	84.13	29.89
	300	739.3	309.5	116.1	48.27
54	30	44.65	30.43	N/A	N/A
	65	92.67	52.52	N/A	N/A
	100	152.5	98.61	N/A	N/A
	200	323.4	164.4	53.13	N/A
	300	535.4	232.9	78.63	30.47
27	30	26.12	20.51	N/A	N/A
	65	53.92	39.16	N/A	N/A
	100	103.2	57.96	27.16	N/A
	200	236.3	120.2	37.47	N/A
	300	384.6	179.7	53.99	N/A

Biology Open • Supplementary information

Table S2. Average pressure values measured in the 20mL syringe held by the clamp mount

Drop height (cm)	Mass of weight (g)	Average maximal pressure (kPa)	Average pressure 1st wave (kPa)	Average pressure 2nd wave (kPa)	Average pressure 3rd wave (kPa)
108	30	50.55	32.83	N/A	N/A
	65	105.3	51.31	N/A	N/A
	100	133.1	73.19	21.88	N/A
	200	286.4	141.3	46.75	13.03
	300	445.3	210.1	66.11	28.32
54	30	45.49	31.20	N/A	N/A
	65	67.40	40.78	N/A	N/A
	100	98.57	56.00	N/A	N/A
	200	185.3	104.3	34.95	10.70
	300	296.1	147.8	60.07	16.92
27	30	24.01	17.01	N/A	N/A
	65	68.24	41.64	N/A	N/A
	100	80.88	47.79	N/A	N/A
	200	120.1	78.79	26.41	13.11
	300	198.8	112.7	42.13	12.68

Biology Open • Supplementary information

Table S3. Average pressure values measured in the 10mL syringe held by the foam block mount

Drop height (cm)	Mass of weight (g)	Average maximal pressure (kPa)	Average pressure 1st wave (kPa)	Average pressure 2nd wave (kPa)	Average pressure 3rd wave (kPa)
108	30	105.3	55.51	N/A	N/A
	65	234.5	91.76	N/A	N/A
	100	459.6	158.4	32.75	N/A
	200	878.9	332.6	119.5	N/A
	300	1105	526.8	227.2	76.58
54	30	83.41	51.00	N/A	N/A
	65	201.8	96.89	N/A	N/A
	100	316.4	132.8	43.93	N/A
	200	630.6	250.6	54.47	N/A
	300	929.3	373.4	167.4	54.51
27	30	33.70	26.16	N/A	N/A
	65	115.0	59.23	N/A	N/A
	100	229.6	112.2	N/A	N/A
	200	410.7	177.3	60.81	N/A
	300	705.6	189.5	109.6	27.82

Biology Open • Supplementary information

Table S4. Average pressure values measured in the 20mL syringe held by the foam block

Drop height (cm)	Mass of weight (g)	Average maximal pressure (kPa)	Average pressure 1st wave (kPa)	Average pressure 2nd wave (kPa)	Average pressure 3rd wave (kPa)
108	30	34.96	23.41	N/A	N/A
	65	128.5	56.68	N/A	N/A
	100	279.3	78.35	43.02	N/A
	200	511.8	157.8	85.33	34.93
	300	678.2	255.1	121.4	59.42
54	30	27.38	20.75	N/A	N/A
	65	70.35	52.34	N/A	N/A
	100	154.6	53.88	N/A	N/A
	200	367.8	116.5	53.90	N/A
	300	482.8	186.9	98.38	46.68
27	30	15.16	11.61	N/A	N/A
	65	40.02	28.10	N/A	N/A
	100	89.73	41.78	N/A	N/A
	200	249.0	89.49	38.54	N/A
	300	323.9	145.8	44.69	N/A

Biology Open • Supplementary information
